# Mechanical and Physical Properties of NaOH-Treated Rubberized Concrete Blocks

**DOI:** 10.1021/acsomega.5c13017

**Published:** 2026-05-19

**Authors:** Víctor Poblete Pulgar, Siva Avudaiappan, Suzanne Segeur Villanueva, Danny Lobos Calquín, Markssuel Teixeira Marvila, Afonso R.G. de Azevedo

**Affiliations:** † Departamento de Ciencias de la Construcción, Facultad de Ciencias de la Construcción y Ordenamiento Territorial, 28092 Universidad Tecnológica Metropolitana, Santiago 8330383, Chile; ‡ Departamento de Planificación y Ordenamiento Territorial, Facultad de Ciencias de la Construcción y Ordenamiento Territorial, Universidad Tecnológica Metropolitana, Santiago 8330383, Chile; § Centro I+D Sustentable, Facultad de Ciencias de la Construcción y Ordenamiento Territorial, Universidad Tecnológica Metropolitana, Santiago 8330383, Chile; ∥ 28109 UENF − State University of the Northern Rio de Janeiro, LECIV − Civil Engineering Laboratory, Campos dos Goytacazes, Rio de Janeiro 28013-602, Brazil

## Abstract

The developments in rubberized concrete technology and its implementation in engineering could greatly facilitate the extensive recycling and proper disposal of waste tire material. The use of rubber crumb in concrete modifies its compressive strength characteristics. The main aim of the proposed study is to determine the mechanical and physical behavior of the rubberized concrete block using NaOH-treated waste tire rubber crumb as a partial replacement aggregate. There were different replacement levels of rubber crumb up to 25% with sodium hydroxide treatment and silica fume to enhance strength performance. Experimental research studies on compressive strength, density, and thermal conductivity. It is found that the higher the content of rubber, the lower will be the compressive strength, but the greater the thermal insulation and the lower the unit weight. A best combination of 15% rubber crumb with silica fume had a compressive strength of 13.91–14.52 MPa, indicating that it could be used in load-bearing masonry construction with lower density. Greater rubber contents exhibited better thermal resistance and could be used as nonstructural and insulation. The findings show that NaOH-treated rubberized concrete can be a sustainable and viable solution to tire waste recycling in construction.

## Graphical abstract

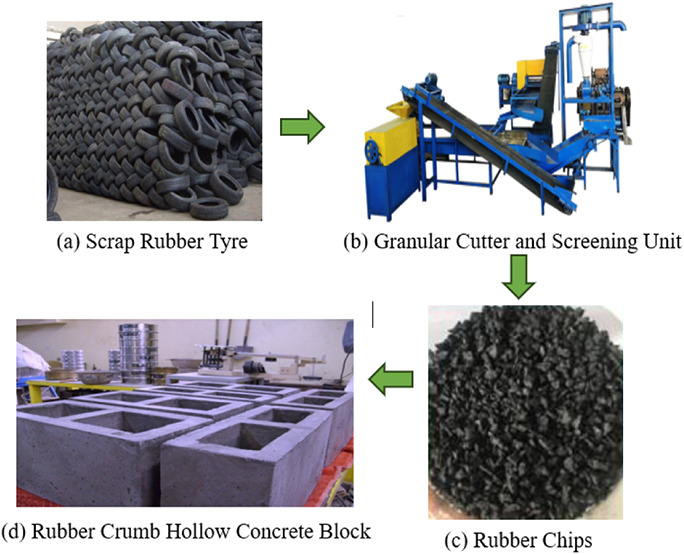

## 1 Introduction

Globally, the improper disposal of waste tire rubber has emerged as a significant environmental concern. Global yearly tire production is projected to be 1.5 billion units.^1,2^ Annually, a vast number of tires are disposed of, or buried worldwide, posing a tremendous ecological hazard. Each year, an estimated 1000 million tires reach their useful life, and among them, over 50% are disposed of in landfills or rubbish without undergoing any form of treatment. By the year 2030, the annual quantity of waste tire would approximate 1,200 million. With the tires stockpiled, there are a total of 5000 million tires that need to be regularly disposed of.^3–5^ The Rubber Manufacturer’s Association^6,7^ publishes statistics indicating that the United States generates about 230 million waste tires annually, with over 75 million of these being stored in stockpiles. When analyzing the Indian situation, it was projected that the annual quantity of wasted tires would reach 112 million after undergoing two rounds of retreading.^8^ Based on estimates from the European Association of rubber tire manufacturers, some 2.9 million tonnes of old tires were disposed of in 2009.^8^ The rate of recovery is 96%, with 17% effectively reprocessed or reused, 39% recycled, and 40% allocated to energy generation.^9^ Waste tires are disposed of using several ways such as landfilling, incineration, fuel utilization, pyrolysis, and carbon black production. Stockpiled tires pose many health, environmental, and economic hazards by potentially contaminating air, water, and soil.^10–12^ Due to their unique form and impermeable nature, the tires provide a breeding ground for mosquitoes and other pests, therefore allowing them to collect water for an extended duration. Burning tires, albeit the most convenient and cost-effective way of disposing of them, poses significant fire risks.^13^ Temperature in that region increases, and the unregulated release of toxic smoke containing potentially hazardous substances poses a significant threat to humans, plants, and animals. Vehicle tires are produced using petrochemical feedstocks, including butadiene and styrene. Combusting tires emits benzene and styrene chemicals. Butadiene is a neoplastic molecule that is liberated from styrene–butadiene polymer under combustion. The air pollutants release particulate smoke of black color that minimizes vision and contaminates painted surfaces. The harmful gas emissions comprise polyaromatic hydro carbons HCl, NO_2_, SO_2_ and CO. The residual powder remaining resulting from combustion contaminates the soil. Pyrolysis has the drawback of generating carbon black powder, which contributes to atmospheric pollution. Economically, the utilization of tire rubber as fuel is not appealing. Carbon black derived from tires is both costlier and of worse quality in comparison to carbon black made from petroleum fuels. A wide range of noncivil engineering and civil applications can utilize rubber tire on road construction, geotechnical works, silo sealing in agriculture, offshore and onshore breakwaters, estuaries to mitigate ship impact, retaining walls in harbors, artificial reefs for enhancing, as cement kiln fuel, source of electricity generation, and aggregate on cement-based products. Yet, millions of tires are being disposed of, discarded, or incinerated worldwide.^14,15^

Concrete is the predominant civil engineering material globally. The present worldwide difficulties include the optimization of expense even though maximizing the durability and strength of concrete, as well as enhancing the environmental sustainability of concrete building. This problem necessitates sophisticated materials capable of substituting the conventional constituents in concrete. Excellent durability, strength, and strain characteristics of tire waste make it a viable alternative to concrete mixtures.^16,17^ Rubber may be included into mortar or concrete either by substituting coarse aggregates or fine aggregates or by serving as a binding agent. The incorporation of rubber crumb (RC) into cementitious composite offers the benefits of reducing CO_2_ content and enhancing environmental sustainability.^18^ Furthermore, the natural sand extraction is altering the course of hydrological flow of river and resulting in the degradation in stability of river bed. The mitigation of impacts might be achieved by conserving river sand by replacing it with RC for purposes in construction. Incorporating flexible rubber with inflexible concrete modifies the general characteristics and performance of concrete^19^ and also has the potential to create lightweight constructions with cost-effectiveness by decreasing the reliance on natural aggregates. A decrease in the unit weight of concrete, ranging from 14% to 28%, can be achieved by substituting 10–30% of sand with Portland cement.^20^ The replacement of natural particles in plain concrete (PC) with rubber often results in a reduction in mechanical strength.^21^ When 5–20% of fly ash in Portland cement is substituted with powdered rubber to create rubberized concrete (RuC), there is a potential decrease in compressive strength ranging from 30% to 63%.^22^ Thomas and Gupta^23^ have determined that substituting 12.5% fly ash (FA) in concrete with cellulose rhizosphere (CR) is the most effective approach for enhancing resistance to water absorption and carbonation while also achieving a modest level of compressive strength. Senin and colleagues^24^ recommended that the rubber percentage in concrete should not exceed 20%. By substituting a small proportion of sand with rubber, RC can achieve superior tensile strength than PC in situations when rubber-concrete adhesion is adequate.^25^ Evidence from most studies indicates that RC has superior ductility, fatigue resistance, and impact resistance compared to PC.^26–28^ The incorporation of rubber can facilitate the consistent and effortless expansion of concrete when subjected to stress.^29^ The application of RC in the fabrication of structural components is done to achieve low density, high durability, and sufficient strength which has been documented.^30^ Further advantageous uses of RC include the mitigation of vibrations in various constructions, decks and bridge walkways, retaining structures, road pavements, industrial floors.^31,32^ RC may also be employed in hydraulic structures, such as dam spillways and tunnels, where extensive resistance to abrasion required;^33^ in acoustic and insulation systems;^34^ in roadside barriers and running tracks, where significant capacity to absorb impact energy is necessary;^35^ in areas of parking;^36^ and in regions of cold climates where there’s significant freeze–thaw forces.^37^ Incorporating tires into concrete may effectively mitigate environmental pollutants.

The experts and academics are now researching the advancement of novel lightweight construction materials composed of mortar with recycled rubber or precast concrete as an environmentally beneficial aggregate.^38^ The application of these aggregates in commonly used precast construction products, such as building bricks and hollow blocks, is investigated. When compared to standard units, these products have shown improved characteristics in terms of thermal and acoustic insulation, as well as resistance to bending and cracking shrinkage.^39,40^ In addition, the use of these rubberized masonry pieces in slabs or walls as vertical facing is a highly effective energy-saving approach since they considerably decrease the yearly energy consumption associated with building maintenance.^41^ While there are significant advantages like health and environmental benefits associated with recycling used tires, utilization of rubberized construction materials is highly restricted due to several reasons.^42^ The addition of rubber aggregates to rubberized concrete leads to a loss in both compressive strength and durability.^43^ This decrease is contingent upon the dimensions of rubber particles included in the mixture.^44^ Two types of size aggregates that may be efficiently achieved by mechanical grinding are coarse and fine aggregates.^45^ However, using the former, often referred to as rubber crumb (CR), appears to be a more favorable option since the decrease in compressive strength is considerably less significant compared to the latter options.^46^ Furthermore, there remains a dearth of detailed knowledge about the intrinsic characteristics and long-lasting nature of rubberized bricks or hollow blocks. There are some published research on this topic, and no specific regulations pertaining to rubberized dry mortars have been authorized so far.^47,48^ This scenario generates ambiguity among makers of masonry goods, which hampers their attempts to become commercially viable.^49^ Empirical investigations have already shown that CR aggregates have an impact on the workability, porosity, and hydration of newly mixed mortar mixes.^50^ Regulation of these characteristics is crucial for the production of practical dry mix mortars. Excessive fluctuations in these parameters may indicate faulty cast goods with substantial volumetric changes and major cracks.^51^ Furthermore, these variances might cause significant changes in the processes of handling of rubberized mortars, which may impact conditions of production or even the geometry of molds. Masonry unit acoustic, thermal, and electrical properties are thus highly dependent on casting mold design. Most molds are provided by substantial hollow spaces and noticeable slenderness in their walls. To prevent collapse of newly manufactured masonry units during shipment or demolding, rubberized mortars must adhere to similar rigorous technical requirements as conventional mortars. Additionally, it is essential to get affordable construction components that may be efficiently and successfully integrated into industrial production processes. Nevertheless, the expensive procedure of producing CR from scrap tires has a detrimental impact on the profitability of rubberized construction material.^52^ While there is a substantial body of research on the characteristics of rubberized precast concrete and mortar, specific investigations on the cost-benefit analysis remain limited. Studies by Amr et al. highlight the potential of self-compacting and roller-compacted recycled crumb rubber rubberized concrete, as it gives better compressive strength.^53^ Previous studies by Ibrahim et al. have demonstrated that incorporating NaOH-treated crumb rubber and recycled steel fibers from waste tires in concrete can enhance tensile and flexural performance under controlled curing conditions. While compressive strength and permeability are influenced by drying regimes, such composites offer an environmentally sustainable alternative for green concrete applications.^54^ Supplementary and more comprehensive investigations are required to ensure the intrinsic characteristics, longevity, and feasibility of these items prior to their integration into contemporary industrial facilities. In fully automated industrial processes, this work investigates the utilization of rubber crumb as a dry-mix mortar aggregate to manufacture bricks or rubberized long hollow blocks. Initially, a sequence of studies was conducted in the laboratory to assess the viability of several mortar mixtures for automated industrial machinery. The fine aggregate was substituted by weight in varying proportions of untreated RC soil. Several combinations were chosen as the most suitable in terms of their workability and compressive strength for the experiments conducted in the plant. The experiments encompassed the industrial manufacturing of rubberized mortar-reinforced hollow blocks and bricks. Both may serve as efficient and ecologically beneficial technologies for the construction of buildings. Dimensional distortions and reduction in compressive strength were quantified. An economic evaluation was conducted at the conclusion of the study to examine the technical viability of the mixtures and ascertain the overall production expenses of the rubberized bricks.

The construction industry has experienced tremendous growth and led to a high demand of concrete just like never before, and at the same time, the construction industry has produced volumes of industrial and municipal waste. Of all these, discarded car tires are a significant environmental problem because they are nonbiodegradable, they have the potential of catching fire, and they cannot be disposed of in landfills. Recycling of waste tires as crumb rubber to be used in construction would offer an opportunity for sustainable construction as well as reducing the waste management challenge. It has been previously demonstrated that the addition of rubber crumb to concrete enhances impact, ductility, and thermal insulation. Nonetheless, its addition also results in a decrease in compressive strength and stiffness with the presence of poor interfacial bonding and the presence of a higher porosity. Surface treatments, e.g., sodium hydroxide (NaOH), are suggested to improve rubber-cement adhesion, whereas the use of additional cementitious materials (e.g., silica fume) may be used to increase matrix density and strength. Although there has been increased attention on rubberized concrete, much of the available research has concentrated on the mechanical strength, whereas little has been done regarding the joint analysis of mechanical performance, density, and thermal conductivity in rubberized concrete blocks that are to be used in a real construction environment. Specifically, the literature on NaOH-treated crumb rubber in combination with silica fume in masonry-grade concrete is not systematically studied, and both structural capacity and thermal efficiency are of great concern.

### 1.1 Problem Statement

Despite the fact that waste tire rubber has a great potential in the sustainable production of concrete, the loss in strength and poor knowledge regarding its impact on thermal and material properties limit its use in practice for bearing and insulation construction.

### 1.2 Research Gap

Experimental evidence of the combined effect of the treatment of NaOH crumb rubber and silica fume on the mechanical, thermal, and density characteristics of rubberized concrete blocks is limited, especially when it comes to the application in the construction of masonry and low-energy consumption. The novelty of this work is introduced by the built-in determination of strength, density, and thermal conductivity of NaOH-treated rubberized concrete using silica fume, giving useful proportions of compositions that could be applied with load-bearing or insulation units of masonry. These two performances are toward achieving energy-efficient and sustainable building materials.

## 2 Source of Waste Tire Rubber

Rubber waste mostly consists of wastes of tire, which is categorized as automotive and truck tires.^55^ The physical characteristics and compositions of tires from different suppliers vary significantly. Therefore, their various applications have distinct impacts on the strength of concrete.^56^ A tire typically consists of synthetic and natural rubbers, textile fabric, metal, carbon black, and additives.^57^ The residual asphalt can be removed from tires through mechanical grinding, cryogenic, or pyrolysis temperatures. The rubber components are vulcanized together to obtain the defined properties of tires. However, the use of different additives like stabilizers, antiozonants, and antioxidants in the manufacturing of rubber tires causes them to become nonbiodegradable and resistant to photochemical breakdown.^58^ Efficient waste tire management poses technological, economic, and ecological challenges. Despite the unique composition of automobile and truck tires, the majority of them have a similar proportion of natural and synthetic rubber components. Rubber may be extracted from several types of tires, ranging from 14% to 55%, depending on the specific compositions. Tire tread and sidewall components contribute the majority of rubber content.

## 3 Extraction of Rubber Crumbs

In the tire life cycle, there are five common phases: extraction, production, utilization, tire collection, and management of the trash tire. Following the pickup of End of Tyres ELT, the subsequent stage entails the process of recovery and landfilling. Landfilling tires presents a significant ecological hazard. Aside from diminishing biodiversity, tire disposal sites predominantly consist of dangerous and easily soluble substances.^59^ The global practice of tire landfilling has been consistently declining, while other methods for recovery exist. These include “energy recovery,” which involves using discarded tires as a substitute for fuels of fossil when the calorific value is equivalent to high-quality coal and “chemical methods” such as thermolysis, pyrolysis, gasification, and polymer recovery. Using large machinery, the latter process involves tire shredding and chipping to separate tires into small pieces of different sizes suitable for various civil engineering applications such as carpets for shock absorbing in sports stadiums or playgrounds, paving blocks, rubberized asphalt pavements, roofing materials, pervious concrete, concrete pavement subgrade fill embankments and highways and other geotechnical uses.^60^ In Figure 1, the process of tire shredding is illustrated. Since waste rubbers do not naturally decompose and crumble, the most cost-effective approach is to incinerate them, resulting in the generation of a substantial amount of smoke. Thus, it is necessary to recycle any remaining rubbers now. Rubberized concrete (Ru) is a concrete produced by incorporating rubber tires. Its flexibility, energy absorption, low weight, and heat-insulating properties have made it a promising material in the construction industry.^61^ Implementing this recycling method in concrete is highly useful for both environmental conservation and energy savings. One metric tonne of rubber crumb-modified asphalt mixture requires 265 MJ less energy to produce. There is a 3.76 kg reduction in CO_2_ emissions (29.79%), almost 62% fewer dangerous emissions of gas is generated, with cost savings of USD 29.00.^62^ The recycling of tires into aggregates for cement concrete involves the shredding of three distinct kinds. The coarse aggregate consists of rubber chips that are produced by two processing methods. Tyre rubber is first shredded into fragments of 350 to 480 mm in length and 120 to 250 mm in breadth. In the second step, particles with sizes ranging from 13 mm to 76 mm are produced. Rubber crumb, which is used as a partial substitute for fine aggregate, is generated by two methods: The particles are produced in the size range of 0.075 mm to 4.75 mm by utilizing cracker mills at ambient temperature and applying liquid nitrogen through a cryogenic process at a temperature below 80 °C.^63^ In the second process of micromilling process, finely ground powder is generated as a very fine aggregate, with particle sizes ranging from 0.5 mm to 0.075 mm. Figure 2 illustrates nonfunctional rubber tires, tire fragments, and residual rubber, correspondingly. Utilizing discarded tire rubber as a substitute for fine and coarse aggregate presents a considerable obstacle in terms of its binding behavior performance inside the cement matrix.^64^ Inadequate adhesion between rubber particles and the cement paste leads to a substantial decrease in its mechanical^65^ and structural qualities.^66^ In order to surmount this obstacle, scholars have explored several methods to enhance the adhesiveness of rubber particles and to enhance the mechanical and durability characteristics of rubber concrete.

**Figure 1:**
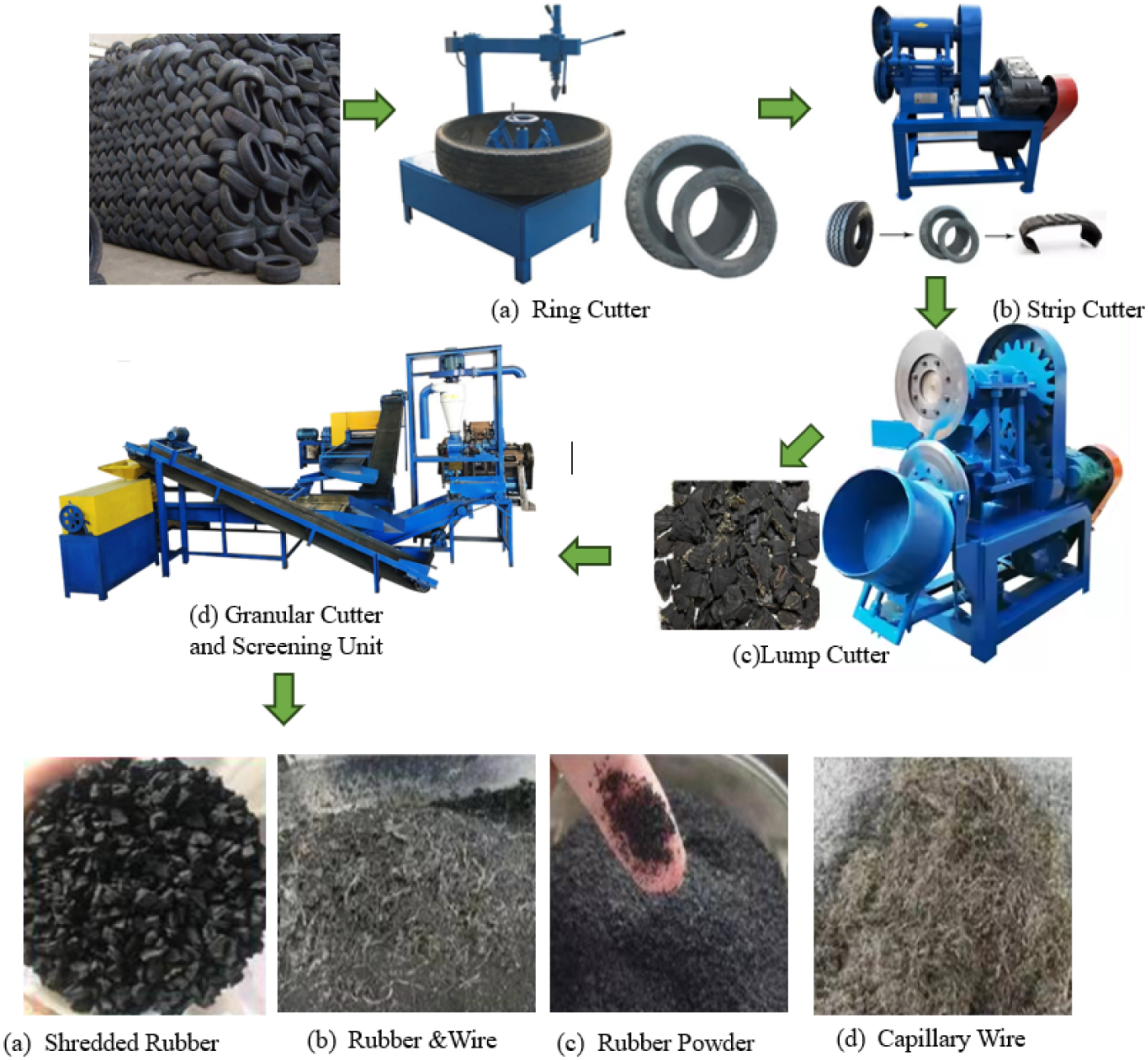
Methodology for the fragmentation of tire waste.

**Figure 2:**
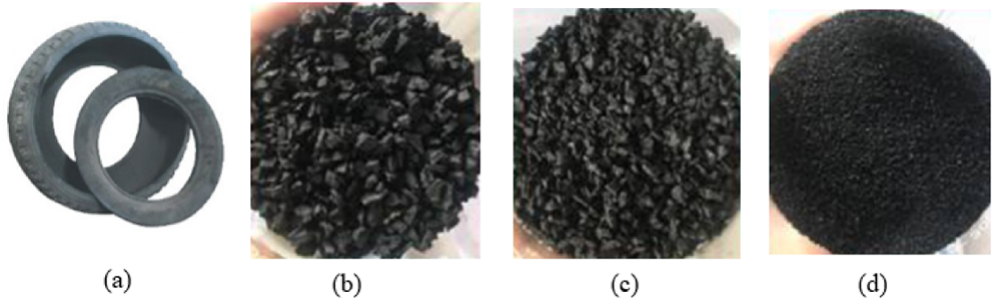
(a) Waste tire, (b) shredded rubber, (c) rubber chips, (d) and rubber crumbs segregation of waste tyre.

## 4 Preparation of Rubber Crumbs

Treatment of rubber particles prior to application can significantly enhance the adhesion of the cement/rubber contact in the matrix of concrete by eliminating contaminants and altering the topography and surface shape. Several pretreatment methods were employed based on the recommendations of other research^67^ to enhance the performance of Rubcrete in several studies. Water washing, water soaking, soaking in NaHSO_4_–KMnO4, H2SO4, CaCl_2_, H_2_O_2_, silane, and NaOH were the methods employed. Table 1 shows the rubber crumb pretreatment methods. Previous studies have shown that the interfacial bonding between crumb rubber and cement paste has been reported to be enhanced with the help of many surface treatment methods.^68,69^ One of the most widely implemented methods is the alkali (NaOH) treatment, which eliminates the surface contaminants, increases the roughness of the surface, and introduces polar functional groups, thus increasing the adhesion of the rubber-cement. Silane coupling agents are more effective as they offer more powerful chemical bonding; that is, they can form covalent connections between rubber and hydration products, but they are very expensive and complicated to apply, which makes it impossible to use these agents on a large scale. Oxidative agents, including hydrogen peroxide and potassium permanganate, raise the particle surface polarity and wettability but can partially degrade rubber and need to be strictly handled. NaOH treatment provides a good economical solution that has good scalability in the production of concrete in comparison with those modes of treatment. In the current experiment, the initial performance of rubberized foam concrete was determined by using untreated crumb rubber to allow the intrinsic changes caused by the addition of rubber to the concrete to be clearly measured without the interference of chemical modification.

**Table 1:** Rubber Crumb Pretreatment Methods

Treatment Method	Chemical/Medium	Concentration	Treatment Duration	Drying Method	Purpose/Effect
Water washing	Tap water	–	5 min (high-pressure rinse)	Air drying	Removes surface dust, dirt and loose contaminants
Water soaking	Tap water	–	24 h soaking + rinsing	Air drying	Removes soluble impurities and softens rubber surface
NaOH treatment	Sodium hydroxide	10%	30 min	Rinsed to pH 7, air-dried	Increases surface roughness and hydrophilicity, improves rubber–cement bond
H_2_O_2_ treatment	Hydrogen peroxide	10%	30 min	Rinsed to pH 7, air-dried	Oxidizes surface, enhances polarity and adhesion
CaCl_2_ treatment	Calcium chloride	10%	Up to 24 h	Air-dried (no rinsing)	Improves surface ionic activity and cement bonding
H_2_SO_4_ treatment	Sulfuric acid	35%	24 h	Rinsed, air-dried	Strong surface etching and roughening
Silane treatment	Silane coupling agent	1%	20 min mixing +30 min heating at 80 °C	Alcohol wash, air-dried	Forms chemical bridge between rubber and cement paste
KMnO_4_ – NaHSO_4_	Potassium permanganate + Sodium bisulfate	5% + 5%	2 h (KMnO_4_ at 60 °C) + 1 h (NaHSO_4_ at 60 °C)	Rinsed to pH 7, air-dried	Sulphonation and oxidation, enhances surface reactivity

The concentration of NaOH used (10%) and the time of treatment (10 min) in this study were chosen as a compromise between the effectiveness of surface modification and the integrity of the material based on the findings of earlier studies done on the topic of rubberized concrete.^70^ Previous literature has indicated that low concentrations of NaOH (under 5% of solution) result in slight surface roughness and not much in improving the bonding of rubber cement, and high levels (above 10–12%) may result in excessive mass degradation, brittle rubber, and mass loss of particles. In the same manner, shorter than 20 min treatment time can be too short to trigger the rubber surface, whereas the longer than 40–60 min can be damaging the polymer structure and lowering the mechanical stability. NaOH solution, 10%, 30 min of immersion has been widely reported as a good range that both removes surface contaminants, increases crumb rubber surface roughness, and increases wettability without reducing mechanical integrity of the crumb rubber. These parameters were hence used in the current paper to attain high interfacial bonding without compromising the strength and elasticity of the rubber particles.

## 5 Materials

### 5.1 Pozzolanic and Cementitious Materials

Portland cement (OPC) type I, which meets the specifications of ASTM C150 (ASTM, 2005f), was utilized to create the mixture of concrete. The experimental investigation employed silica fumes acquired from Astra Chemicals. The primary objective of utilizing silica fume is to improve the adhesion between the rubber crumb particles and the cement paste in the concrete mix. Silica fume’s specific gravity and chemical composition were determined following the specifications of ASTM C311 and ASTM C1240. The specific gravity of cement was calculated using the ASTM C188 specifications. The chemical compositions of silica fume and cement are presented in Table 2.

**Table 2:** Chemical Composition of Cement and Silica Fume

Chemical Composition	MgO	SO_3_	Fe_2_O_3_	Al_2_O_3_	SiO_2_	CaO	Na_2_O	K_2_O	Specific gravity
Cement	1.08	2.18	3.63	5.32	21.54	63.33	-	-	3.2
Silica Fume	0.061	-	0.421	0.065	92.22	0.073	0.029	0.268	2.24

### 5.2 Coarse Aggregate, Fine Aggregate, and Rubber Crumb

Rubber crumb and river sand, of size passing through 600 mm, and gravel of 10 mm nominal size were utilized as aggregate in the reinforced concrete mixes. Rubber crumb, fine aggregate, and oven-dry density are tested in accordance with the standards set out by ASTM C128 and ASTM C127, respectively. Table 3 shows the test results. Figure 3 displays the grading of river sand, coarse aggregate, and rubber crumb. The river sand and coarse aggregate sieve analyses were conducted in compliance with the standards laid out in ASTM C136.

**Table 3:** Properties of Fine Aggregate, Coarse Aggregate and Rubber Crumb

Properties	Fine aggregate	Coarse aggregate	Rubber crumb
Moisture content, %	16.7	0.94	1.15
Water absorption, %	4.48	1.13	-
Specific gravity	2.71	2.65	0.95
Fineness modulus	2.32	-	0.92

**Figure 3:**
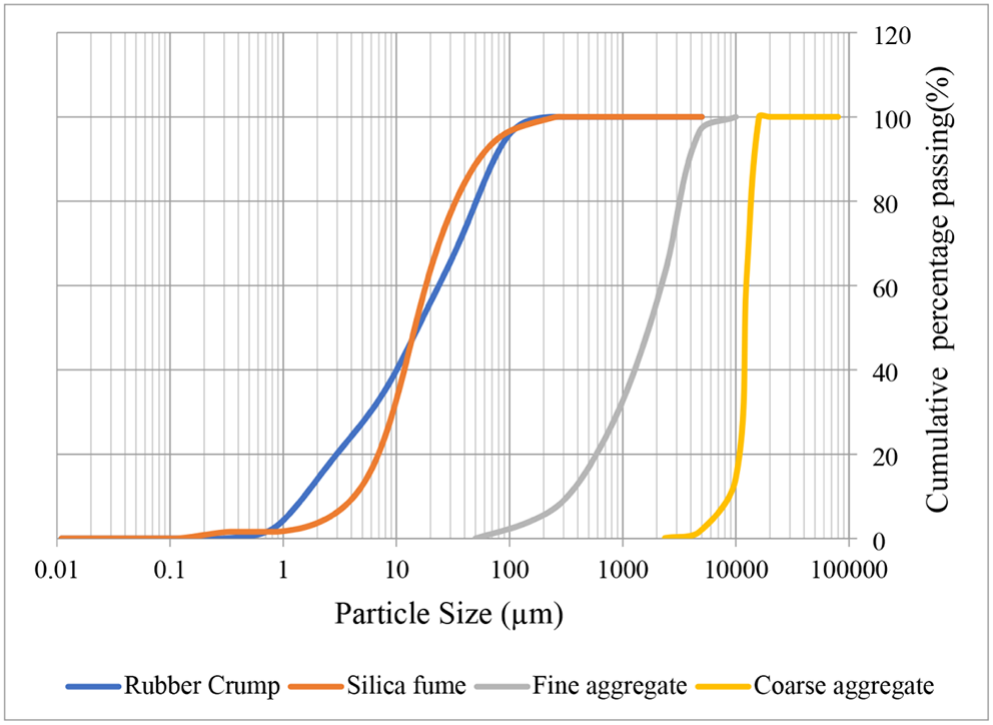
Particle size distribution of fine, coarse aggregate, silica fume, and rubber crumb.

## 6 Mixture Proportions

This study involved preparing 16 different concrete mixes incorporating rubber crumb as a partial replacement for fine aggregate and silica fume as a partial replacement for cement. The conventional mix ratio by volume cement: fine aggregate: coarse aggregate was used throughout the study. The RC replacement levels varied from 0% to 30% by volume, while the SF replacement levels were set at either 0%, 3%, or 4%, as shown in Table 4. The mixes were molded and tested for compressive strength to assess their suitability for structural applications. Eight percent water content was used for each batch of mix, in line with the practices of hollow concrete block manufacturers. This slightly higher water-to-cement ratio ensured adequate workability and strength development in zero-slump concrete mixtures. The optimum level of inclusion of SF was selected based on the results of the compressive strength test conducted on previous research and trial mixtures prepared with SF.^71^ The results showed that the compressive strength varied across the different mixtures, with some achieving the requirements of the BS EN 771 and ASTM C90 standards for concrete blocks. Out of the 16 mixes, the best were selected based on mechanical strength for further investigation. The optimization of RC and SF content aimed to enhance the mechanical properties of the concrete while maintaining the required standards for load-bearing applications. The study demonstrated that careful proportioning and selection of materials are crucial in developing concrete mixes that meet industry standards, highlighting the potential for rubber crumbs as a viable partial replacement for fine aggregates in concrete production.

**Table 4:** Mix Proportions of Rubber Crumb Hollow Concrete Block

Mix	Strength	Cement	Silica	FA	CA	RC
	MPa	%	%	%	%	%
1	6.03	6	4	30	60	0
2	9.21	6	4	30	60	5
3	12.65	6	4	30	60	10
4	13.91	6	4	30	60	15
5	14.85	6	4	30	60	20
6	10.52	6	4	30	60	25
7	7.04	6	4	30	60	30
8	7.52	7	3	30	60	0
9	10.21	7	3	30	60	5
10	13.12	7	3	30	60	10
11	14.52	7	3	30	60	15
12	16.12	7	3	30	60	20
13	14.98	7	3	30	60	25
14	11.12	7	3	30	60	30
16	18.63	10	0	30	60	0

### 6.1 Sample Preparation for Testing

The specimens for the strength test of compression are cast following the stipulated criteria of BS EN 12390-2. Adjustments were made to the compaction technique (rod tamping) in order to meet the specifications of the hollow concrete block manufacturers. Within the factory, hollow concrete blocks were manufactured by applying vibration and pressure to the mold in order to minimize the void ratio. The concrete placed in the mold was compacted in three successive layers, with each layer being uniformly tamped to ensure consistent densification for removing entrapped air. Compaction was achieved by applying a compression force of 600 kN min^1^ for 1 min using a compression machine to compact the material in the mold. The test samples were unmolded after 24 h and then underwent water curing.

### 6.2 Production of Rubber Crumb Hollow Concrete Block (RCHCB) in the Laboratory

The RCHCB depicted in Figure 4 was manufactured using molds with internal dimensions of 390 mm × 190 mm × 190 mm (length, width, and depth), two hollow cavities, and 75% of the total volume. Upon completion of compaction, RCHCBs were promptly extracted from the steel mold (to replicate the standard production process) and allowed to freeze at ambient temperature for 24 h. Water curing was thereafter performed for a duration of 28 days prior to experimental testing.

**Figure 4:**
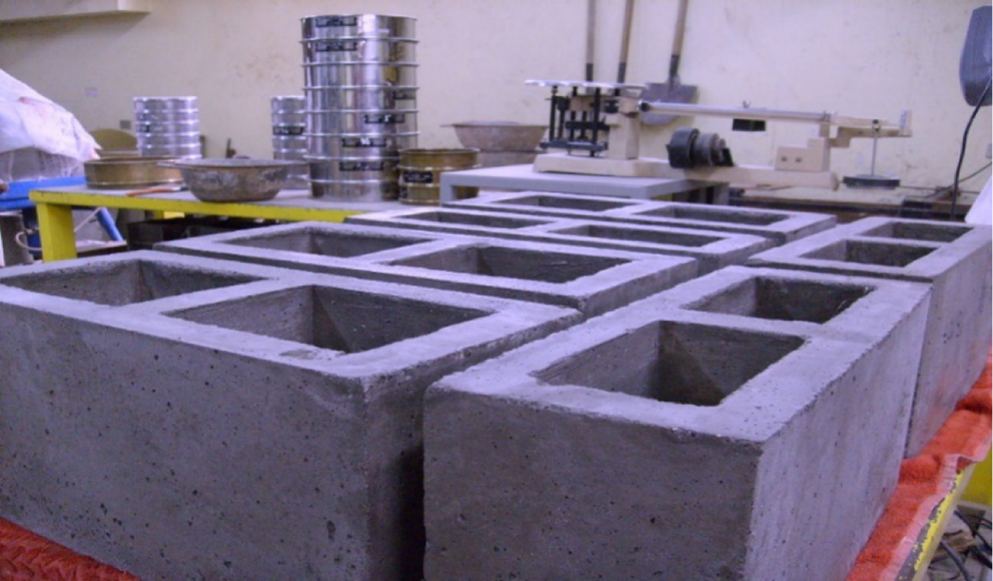
Production of rubber crumb hollow concrete block.

## 7 Test Procedures

### 7.1 Compressive Strength and Density for RCHCB

For the RCHCB, the water absorption, density, and compressive strength tests were conducted following the specifications of ASTM C140. The compressive strength of the RCHCB was measured for 28 days using universal testing equipment fitted with stiff steel plates measuring 200 mm × 400 mm. The steel plates have dimensions that are a minimum of 5 mm larger than the dimensions of the RCHCB units, as specified in ASTM C140. All specimens of the RCHCB were prepared by saw-cutting to provide a flat surface for testing.

### 7.2 Evaluation of Thermal Conductivity

Linear heat conductivity refers to the process of transfer of heat within a substance. The technique, called the steady-state approach, was first proposed by Tritt. The heat conductivity rate was determined using eq 1 following the ASTM C1045 guideline.

1 $$\lambda =\frac{QL}{A\left(T_{h}-T_{c}\right)}$$

In this equation, the rate of one-dimensional heat flow across the metering area is represented by Q, and it is measured in time. A is the area of the transfer surface for heat, L is the material’s thickness, which determines how much heat is transmitted. The thermal conductivity, denoted by *λ*, is temperature- and material-dependent, and the surface temperatures of the specimen are denoted as Th and Tc, which are hot and cold, respectively. The thermal conductivity was calculated using eq 1 based on ASTM C1045, which relates steady-state heat flow through the specimen to the temperature difference across it. Here, *Q*is the heat flow rate, *L*is specimen thickness, *A*is the heat transfer area, and *T*_*h*_ – *T_c_* is the temperature gradient. The equation quantifies the material’s resistance to heat flow.

## 8 Results and Discussion

### 8.1 Compressive Strength and Density of Rubber Crumb Hollow Concrete Block

Compressive strength and density of rubber crumb hollow concrete blocks with various mixtures of crumb rubber and silica fume are provided in Table 5. Silica fume plays a critical role in enhancing the mechanical performance and structural integrity of concrete hollow blocks by modifying the interfacial transition zone (ITZ) between the cement paste and aggregates.^72^ When silica fume is introduced into the concrete mix, it undergoes a pozzolanic reaction with calcium hydroxide (CH) produced during cement hydration. This reaction generates additional calcium silicate hydrate (C–S–H), which is the primary compound responsible for concrete’s strength and durability.^73^ The ITZ, typically the weakest region in concrete, benefits significantly from the introduction of silica fume. Silica fume particles are smaller than cement particles, enabling them to fill the microscopic pores in the ITZ, leading to a denser microstructure. Moreover, interfacial binding between the rubber crumb and the cementitious matrix is also greatly improved with the surface treatment of rubber particles with NaOH. The alkaline treatment eliminates surface impurities like zinc stearate and other hydrophobic contaminants and causes surface roughness and the creation of microtextures on the rubber surface. This change enhances the wettability and provides high mechanical interlocking between rubber particles and the cement paste that surrounds them Also, silica fume plays a complementary role in the refinement of the interfacial transition zone (ITZ). Because of its ultrafine particle size and high pozzolanic reactivity, silica fume occupies micro-voids and capillary pores in the ITZ, resulting in a more dense microstructure. The pozzolanic reaction of silica fume and calcium hydroxide also results in secondary calcium silicate hydrate (C5H), which improves the bond strength at the interface. The overall effect of NaOH treatment plus the addition of silica fume is less interfacial porosity, low microcrack initiation, and high stress transfer effectiveness between the rubber particle and cement matrix, which leads to enhanced mechanical performance in moderate levels of rubber replacement. This densification reduces porosity, narrows the ITZ thickness, and significantly strengthens the bond between the cement paste and the aggregates. Additionally, the refinement of the ITZ by silica fume reduces the occurrence of microcracks, thereby improving the overall structural integrity of the concrete matrix and increasing compressive strength. A comparison of different mix designs, each containing various proportions of cement, silica fume, and rubber crumb (RC), illustrates how these components influence the compressive strength and density of concrete hollow blocks. For example, Mix C6RC0, which contains no RC but 6% cement and 4% silica fume, exhibits a compressive strength of 6.03 MPa and a density of 1735 kg/m^3^. The absence of RC results in a relatively high density because the block lacks the lightweight properties introduced by CR particles. However, when 15% RC is added, as in Mix C6RC15 (6% cement and 4% silica fume), the strength increases to 13.91 MPa, while the density decreases to 1598 kg/m^3^. The reduction in density can be attributed to the lower specific gravity of RC (0.95) compared to fine aggregates (2.57). In another mix, C7RC15, where the cement content is increased to 7% and silica fume reduced to 3%, the compressive strength further improves to 14.52 MPa, and the density decreases to 1495 kg/m^3^. The additional cement content promotes the formation of more C–S–H, compensating for the reduction in silica fume and contributing to enhanced structural properties. Conversely, Mix C10RC0, which contains 10% cement and no silica fume or RC, achieves the highest strength of 18.63 MPa and a density of 1821 kg/m^3^. The high cement content generates abundant C–S–H, leading to superior strength, though the density remains relatively high due to the absence of RC. On the other hand, Mix C6RC30, which contains 30% RC, 6% cement, and 4% silica fume, experiences a significant reduction in both strength and density. This mix exhibits a compressive strength of 7.04 MPa and a density of 1364 kg/m^3^ as shown in Figures 5 and 6. The high RC content reduces the density due to the lightweight nature of RC, but it also weakens the bonding between the RC particles and the cement paste, resulting in microcracks and a reduction in compressive strength. Silica fume, although improving the ITZ, cannot fully offset the loss in strength caused by the increased RC content. The strength trend indicates that as RC content increases, the compressive strength generally decreases due to the weak bond between the cement paste and RC particles. Silica fume mitigates this loss by refining the ITZ, but its effectiveness diminishes as RC content increases. The density trend shows a consistent decrease with higher RC content, which is due to the lower specific gravity of RC compared to fine aggregates. Silica fume also contributes slightly to reducing density because of its lower specific gravity relative to cement. For load-bearing applications, mixes like C6RC15 and C7RC15 offer an optimal balance between strength and reduced density, whereas for nonload-bearing applications, mixes with higher RC content, such as C6RC30, may be preferable due to their lower density, despite the reduction in compressive strength. The observed nonmonotonic tendency of slightly greater compressive strength of C6CR20 than C6CR15 is due to improved particle packing and a more effective interfacial transition zone (ITZ) due to the combined action of silica fume and rubber crumb distribution. Silica fume fills the ITZ and minimizes the micro-voids.

**Table 5:** Results of Rubber Crumb Hollow Concrete Block

Mix	Mix ID	Strength (MPa)	Cement (%)	Silica (%)	FA (%)	CA (%)	CR (%)	Density (kg/m3)	Thermal Conductivity (W/m.K)
1	C6CR0	6.03	6	4	30	60	0	1735	1.5
2	C6CR5	9.21	6	4	30	60	5	1699	1.43
3	C6CR10	12.65	6	4	30	60	10	1635	1.35
4	C6CR15	13.91	6	4	30	60	15	1598	1.27
5	C6CR20	14.85	6	4	30	60	20	1526	1.20
6	C6CR25	10.52	6	4	30	60	25	1421	1.12
7	C6CR30	7.04	6	4	30	60	30	1364	1.05
8	C7CR0	7.52	7	3	30	60	0	1782	1.55
9	C7CR5	10.21	7	3	30	60	5	1675	1.48
10	C7CR10	13.12	7	3	30	60	10	1578	1.40
11	C7CR15	14.52	7	3	30	60	15	1495	1.33
12	C7CR20	16.12	7	3	30	60	20	1421	1.25
13	C7CR25	14.98	7	3	30	60	25	1354	1.18
14	C7CR30	11.12	7	3	30	60	30	1311	1.10
16	C10CR0	18.63	10	0	30	60	0	1821	1.60

**Figure 5:**
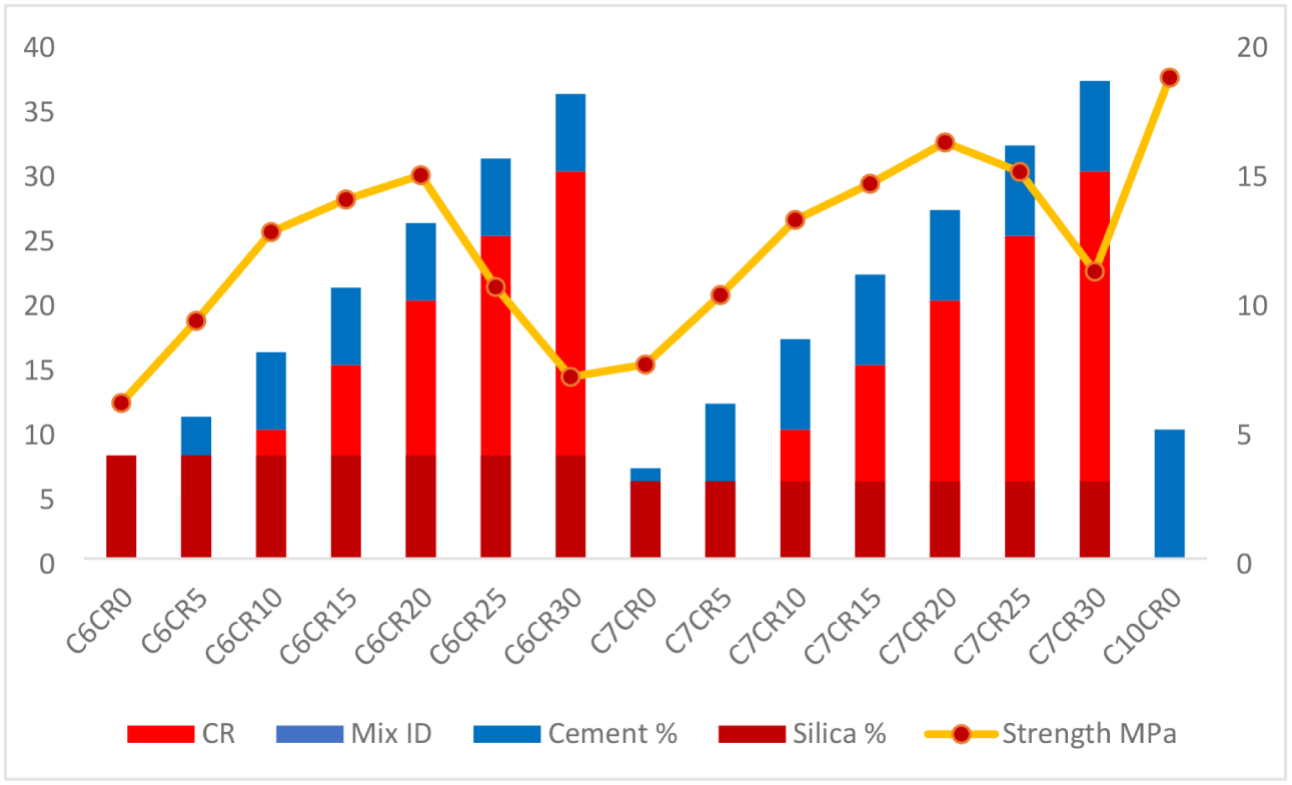
Rubber crumb hollow concrete block compressive strength.

**Figure 6:**
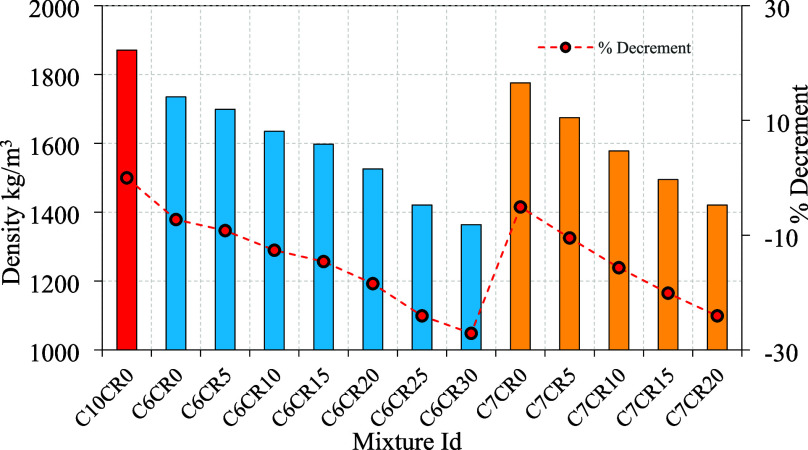
Rubber crumb hollow concrete block density.

Figure 5 shows that compressive strength initially increases for mixes containing up to 10–15% rubber crumb with silica fume due to improved packing and bonding. Beyond this level, strength decreases because of increased porosity and weaker cement–rubber interaction, indicating an optimum rubber content for strength performance

Figure 6 shows a progressive reduction in density with increasing rubber crumb content, reflecting the lower specific gravity of rubber compared to that of natural aggregates. The density decrease is more pronounced at higher replacement levels, leading to lighter blocks suitable for nonstructural and insulation applications. The percentage decrement curve confirms that rubber incorporation significantly reduces the unit weight while maintaining acceptable density ranges for masonry blocks.

### 8.2 Thermal Conductivity

The thermal conductivities of the RC mixtures are shown in Figure 7, with results indicating that thermal conductivity decreases as the percentage of RC (rubber crumb) replacement increases. This reduction can be attributed to the unique microstructure of RC, where air is trapped on the surface of RC particles. The presence of these air voids increases the overall air content within the concrete, significantly impacting its thermal properties. Air, with a thermal conductivity of 0.025 W/m·K, is much less conductive than conventional concrete, which typically has a thermal conductivity of around 1.7 W/m·K. Consequently, the air voids within the concrete matrix create a barrier to thermal transfer, reducing the overall thermal conductivity of the concrete.

**Figure 7:**
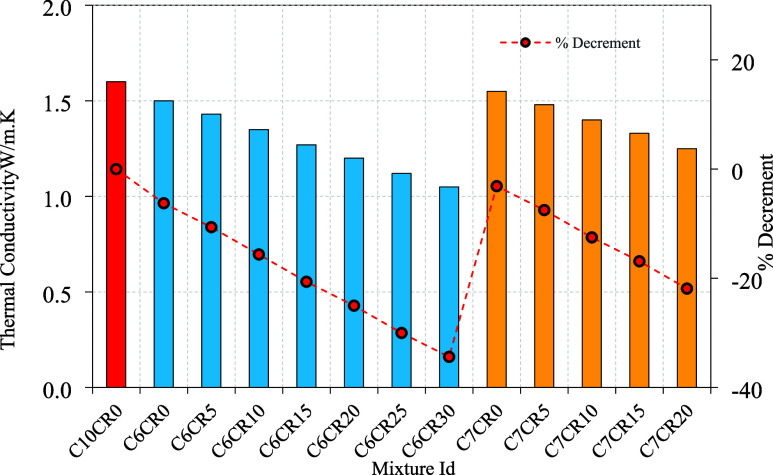
Rubber crumb hollow concrete block thermal conductivity.

Additionally, RC particles themselves have a lower thermal conductivity (0.16 W/m·K) compared to traditional fine aggregates, which typically have a thermal conductivity of about 1.5 W/m·K. This further contributes to the reduction in thermal conductivity as RC content increases. For example, in mixtures with 0% RC replacement, such as Mix C6CR0, the thermal conductivity is 1.50 W/m·K. However, as the RC replacement increases to 5%, 10%, and 15%, the thermal conductivities decrease to 1.43 W/m·K, 1.35 W/m·K, and 1.27 W/m·K, respectively, as shown in Figure 7. The trend continues with higher RC replacements, with Mix C6CR20 showing a thermal conductivity of 1.20 W/m·K, and Mix C6CR30 showing the lowest at 1.05 W/m·K. This pattern is consistent across the mixtures, demonstrating the influence of RC on thermal resistance.

The inclusion of silica fume, used as a partial replacement for cement, also contributes to the reduction in thermal conductivity. Silica fume has a lower thermal conductivity compared to cement, which means that its addition not only enhances the concrete’s strength properties but also aids in further lowering thermal conductivity. This is evident in the mixtures with higher cement content, such as Mix C7RC0 with 7% cement and 3% silica fume, which has a thermal conductivity of 1.55 W/m·K. As the RC replacement in these mixes increases, the thermal conductivity progressively decreases, following a similar trend to the other mixtures. For instance, Mix C7RC10 with 10% RC replacement shows a thermal conductivity of 1.40 W/m·K, while Mix C7RC30 with 30% RC replacement shows a thermal conductivity of 1.10 W/m·K. Compared to conventional concrete, which typically has a thermal conductivity of about 1.6–1.8 W/m·K, the rubber-crumb-modified concrete in this study exhibits significantly improved thermal insulation. The control mix showed a value of 1.50 W/m·K, while increasing rubber crumb replacement reduced it to as low as 1.05 W/m·K at 30% replacement, highlighting the effectiveness of rubber particles and entrapped air in lowering heat transfer.

Overall, the combination of trapped air, the low thermal conductivity of RC, and the incorporation of silica fume effectively reduces the thermal conductivity in concrete mixtures. This makes these mixtures more effective in applications where thermal insulation is desired, leveraging the reduced thermal flow properties of the modified concrete matrix. Similar findings on the impact of silica fume on thermal conductivity were reported in previous studies, such as by Demirboga (2003), highlighting the effectiveness of these modifications in enhancing the thermal properties of the concrete.

## 9 Conclusion

This study evaluated the effects of incorporating rubber crumb (RC) and silica fume (SF) into concrete mixtures, specifically focusing on their impact on compressive strength, thermal conductivity, and density. The following key conclusions were drawn:

- The results indicated that the compressive strength of the concrete mixtures generally decreased as the percentage of RC replacement increased due to the weak bonding between the cement paste and RC particles. However, the partial replacement of cement with silica fume enhanced the compressive strength, with the optimum SF replacement level identified as 3% for mixtures aimed at structural applications.
- The thermal conductivity of the rubber crumb concrete mixtures decreased with increasing RC content. This reduction is attributed to the trapped air on the RC surface and the inherently low thermal conductivity of RC compared to traditional fine aggregates. The addition of silica fume also contributed to lowering thermal conductivity, making these mixtures suitable for applications requiring improved thermal insulation. Increasing RC content led to a decrease in the density of the concrete mixtures, owing to the lower specific gravity of RC compared to fine aggregates.
- The study identified the mix proportions that balanced strength, thermal properties, and other performance criteria. For load-bearing applications, a RC replacement of up to 15% was found to be optimal, whereas mixtures with higher RC content could be suitable for nonload-bearing applications.

On the basis of the combined mechanical, thermal, and density performance, the study determines explicit application-specific optimum mix proportions. High load-bearing and semistructural elements up to 15% rubber crumb with 3% silica fume are recommended in which sufficient compressive strength should be maintained and better thermal efficiency obtained.The decision of rubber contents to be used in nonload-bearing components like partition walls, insulation sheets, lightweight blocks, and roofing panels, where low density and thermal conduction are more important than strength, is that of higher contents. Sustainability wise, the waste tire rubber is a much cheaper material since it can reduce landfill disposal and save on natural sand, and silica fume can increase the material strength and durability which makes it a more attractive material environmentally and economically. To promote the safe use of the rubber-silica fume concrete in the real construction work, future studies should concentrate on the long-term durability, such as freeze–thaw resistance, performance under fire and high temperature, moisture transport, aging, and large-scale structural testing and life-cycle assessment.
